# Effect of Soil pH Increase by Biochar on NO, N_2_O and N_2_ Production during Denitrification in Acid Soils

**DOI:** 10.1371/journal.pone.0138781

**Published:** 2015-09-23

**Authors:** Alfred Obia, Gerard Cornelissen, Jan Mulder, Peter Dörsch

**Affiliations:** 1 Department of Environmental Sciences (IMV), Norwegian University of Life Sciences (NMBU), Ås, Norway; 2 Norwegian Geotechnical Institute (NGI), Department of Environmental Engineering, Ullevål Stadion, Oslo, Norway; 3 Department of Applied Environmental Sciences (ITM), Stockholm University, Stockholm, Sweden; USDA-ARS, UNITED STATES

## Abstract

Biochar (BC) application to soil suppresses emission of nitrous- (N_2_O) and nitric oxide (NO), but the mechanisms are unclear. One of the most prominent features of BC is its alkalizing effect in soils, which may affect denitrification and its product stoichiometry directly or indirectly. We conducted laboratory experiments with anoxic slurries of acid Acrisols from Indonesia and Zambia and two contrasting BCs produced locally from rice husk and cacao shell. Dose-dependent responses of denitrification and gaseous products (NO, N_2_O and N_2_) were assessed by high-resolution gas kinetics and related to the alkalizing effect of the BCs. To delineate the pH effect from other BC effects, we removed part of the alkalinity by leaching the BCs with water and acid prior to incubation. Uncharred cacao shell and sodium hydroxide (NaOH) were also included in the study. The untreated BCs suppressed N_2_O and NO and increased N_2_ production during denitrification, irrespective of the effect on denitrification rate. The extent of N_2_O and NO suppression was dose-dependent and increased with the alkalizing effect of the two BC types, which was strongest for cacao shell BC. Acid leaching of BC, which decreased its alkalizing effect, reduced or eliminated the ability of BC to suppress N_2_O and NO net production. Just like untreated BCs, NaOH reduced net production of N_2_O and NO while increasing that of N_2_. This confirms the importance of altered soil pH for denitrification product stoichiometry. Addition of uncharred cacao shell stimulated denitrification strongly due to availability of labile carbon but only minor effects on the product stoichiometry of denitrification were found, in accordance with its modest effect on soil pH. Our study indicates that stimulation of denitrification was mainly due to increases in labile carbon whereas change in product stoichiometry was mainly due to a change in soil pH.

## Introduction

Denitrification, the microbially mediated, stepwise reduction of nitrogen oxides to N_2_ via nitric oxide (NO) and nitrous oxide (N_2_O) [1], is the dominant pathway returning reactive nitrogen to the atmosphere. NO and N_2_O are gaseous intermediates of denitrification which, once escaped to the atmosphere, have adverse effects on plant and animal health [2], stratospheric ozone [3] and the radiative balance of the Earth [4]. About 45% of the total terrestrial N_2_O emissions can be attributed to nitrogen (N) cycling in agriculture [5], making denitrification a primary target for greenhouse gas abatement [6].

Numerous studies have shown that biochar (BC), a biomass pyrolysis product originally devised for carbon (C) sequestration and soil amelioration [7–10] suppresses N_2_O emissions ([11] and references therein) alongside with increasing crop production [12–14]. Only few studies have reported that BC leads to increased N_2_O emissions [15, 16]. Thus, BC appears to be a promising agent to mitigate N_2_O emissions from agroecosystems, but the mechanisms mediating the suppression are unresolved. Various mechanisms have been proposed, such as increased N_2_O reductase activity at raised soil pH [11], increased electron flow to N_2_O through BC-mediated electron shuttling [17], reduced rates of denitrification through competition for electrons [18], direct sorption of N_2_O [19], improved soil aeration [20] and immobilization of ammonium or nitrate through adsorption or enhanced soil cation/anion exchange [15, 21, 22]. Other proposed mechanisms are ethylene production by BC resulting in temporary inhibition of nitrification [23] and microbial N immobilization due to the presence of labile organic carbon in BC [24]. Increased N_2_O emission after BC application has been attributed to high N content in certain BC such as that made from poultry manure [16, 22].

Most BCs are alkaline owing to their ash content, causing release of base cations, and alkaline properties of organic functional groups [25]. Biochar addition to soils neutralizes soil acidity and may increase the cation exchange capacity (CEC) and base saturation, depending on the intrinsic properties of the soil and the BC [26, 27].

Soil pH strongly controls the N_2_O/(N_2_O+N_2_) product ratio of denitrification. This has been demonstrated for pure cultures of denitrifying bacteria [28] and for soil denitrifying communities [29–33]. The likely reason is that low pH prevents the assembly of functional N_2_O reductase (N_2_OR), the enzyme reducing N_2_O to N_2_ in denitrification [29, 34]. Since BC is generally alkaline, increased N_2_OR activity due to pH rise could be one of the major mechanisms behind the observed suppression of N_2_O emission in BC treated acid soils. If so, N_2_O suppression by BC would be mainly a “liming effect”.

The objectives of the present study were to evaluate the role of BC-induced pH change on NO and N_2_O net production in soil denitrification. Although, NO is an important regulator in many biological processes including denitrification [35, 36], only few BC studies have addressed NO [37]. We carried out *ex situ* denitrification experiments in closed bottles with two acidic agricultural soils from Indonesia and Zambia. We applied increasing doses of two types of BC strongly differing in amount and type of alkalinity and studied the responses of soil pH, overall denitrification rate and gaseous reaction products (NO, N_2_O, N_2_). To shed light on the role of soil pH, we removed alkalinity from the BCs through leaching with water and acid prior to incubation in a second experiment. In a third experiment, sodium hydroxide (NaOH) was used as an alkali analogue to study the effect of pH *per se* in the absence of BC. Furthermore, the NO and N_2_O suppressing effect of BC was compared to that of uncharred feedstock. The denitrification kinetics were studied in stirred soil slurries in helium (He) atmosphere, using a high-throughput incubation robot which monitors oxygen (O_2_), carbon dioxide (CO_2_), NO, N_2_O and N_2_ at high temporal resolution [38]. Stirring ensured homogeneous soil slurries and equilibrium of gases between headspace and liquid phase. Unlike previous studies, our investigations were carried out under fully anoxic conditions, preventing confounding effects on denitrification NO and N_2_O production by other N-cycling processes.

## Materials and Methods

### Soils and biochars

Acidic, sandy loam Acrisols were sampled at Lampung (Sumatra, Indonesia; 05°00.406' S; 105°29.405' E) and Mkushi (Zambia; 13°36.264′ S; 29°29.768′ E) in October 2012 and April 2013, respectively. The soils were sampled from private lands with permission of the owners during the dry season and stored air-dried. Selected soil and BC properties are presented in Table 1. Different N-forms in soils and BCs were not considered. The NH_4_^+^ content was deemed irrelevant because our main experiments were under anaerobic conditions ruling out nitrification. The added ample amount of NO_3_^-^ would override any sorption effect and denitrification and its product stoichiometry, are not sensitive to small differences in NO_3_^-^ availability [39].

**Table 1:** Selected soil and biochar properties^1^.

Soil/Biochar	pH	TN	TOC	TH	H/C	LOI	Ash	Surface area	CEC and base cations (cmol_c_ kg^-1^ soil or char)				
	H_2_O	(%)	(%)	(%)		(%)	(%)	BET m^2^ g^-1^	CEC	K^+^	Na^+^	Ca^2+^	Mg^2+^
Lampung soil	4.0	0.1	1.2	-	-	-	-	-	9.7	<0.1	<0.1	0.3	0.1
Mkushi soil	4.0	0.00	0.5	-	-	-	-	-	6.4	<0.1	0.0	0.1	<0.1
Untreated rice husk BC	8.4	0.9	44.6	1.9	0.51	55.6	51.0	76.4	20	9.5	0.2	3.2	3.6
Water leached rice husk BC	8.2	1.0	48.0	2.1	0.53	59.2	-	108.2	-	-	-	-	-
Acid leached rice husk BC	2.5	0.9	47.8	1.9	0.48	58.2	-	88.5	-	-	-	-	-
Untreated cacao shell BC	9.8	1.5	54.3	1.4	0.31	68.1	18.9	30.9	197	127	0.3	37.1	32.8
Water leached cacao shell BC	9.6	1.8	70.9	1.7	0.29	85.0	-	255.8	-	-	-	-	-
Acid leached cacao shell BC	8.0	1.7	75.9	1.8	0.28	86.7	-	274.8	-	-	-	-	-
Uncharred cacao shell	-	1.4	46.5	-	-	90.3	-	-	-	66.5	0.3	36.7	31.7

^1^TN = Total nitrogen, TOC = Total organic carbon, TH = Total hydrogen, H/C = molar ratio, LOI = Loss on ignition. Untreated BC properties (Ash, CEC & base cations) and surface area data were obtained from Martinsen, Alling [41] and Smebye, Alling [42] respectively. All the other soil and BC data were measured in sub-samples from homogenized bulk samples used in the study. Soil and BC pH was measured in a 1:2.5 v/v slurry in water (n = 2) using a pH meter (Orion 2 Star, Thermo Fisher Scientific, Fort Collins, CO, USA) after overnight sedimentation and shaking. Base cations were measured in the eluate of ammonium acetate at pH 7 for BC and ammonium nitrate for soil (n = 1), with a flame spectrophotometer (Perkin Elmer, AAS 3300). CEC was determined as sum of base cations and exchangeable acidity in ammonium acetate pH 7 and ammonium nitrate extract for BCs and soil respectively. TOC, TN and TH were determined using CHN analyzer (n = 1) (CHN-1000, LECO, USA). The TOC for BCs were determined after acidification to remove carbonates.

The BCs were prepared from rice husk and cacao shell, two common agricultural wastes in Lampung, Indonesia. The two BCs differed in extent and type of alkalinity (Table 1); cacao shell BC had a lower ash content but a ~10 times higher CEC than rice husk BC. The exchangeable cations of cacao shell BC were dominated by potassium (K). Overall, cacao shell BC had a ~5 times higher acid neutralizing capacity (ANC) than rice husk BC (217 vs 45 cmol_c_ kg^-1^) [40].

The BC pyrolysis conditions, taken from Hale, Alling [43], can be found in Description A in S1 File. Since the BCs were not produced in the laboratory, thermogravimetric analyses (TGA) was used to estimate the pyrolysis temperature, indicating that this was between 400 and 500°C. In short, during the TGA, the temperature was stepwise increased up to 900°C, and weight loss was monitored. The weight loss profile was then compared to three temperature series of laboratory-generated BCs where pyrolysis had taken place at an exactly measured temperature. Weight loss and high to low temperature weight loss ratios of our BC samples both showed pyrolysis temperature of 400–500°C.

The BCs used in this experiment were either untreated or leached with water or acid. Leaching of the BCs to partly remove their alkalizing effect before use in the experiments was done on the size fraction ≤ 2 mm. For leaching, columns of 5 cm diameter and 30 cm length were filled with BC. The columns were fitted with tubing at the inlet and outlet and filter paper (0.45μm) was placed on both ends of the column. Biochars were first leached with demineralized water at a 1:50 (BC:water w/w) ratio with a flow rate of 70–80 ml hr^-1^ for 4 days to produce “water-leached” BC. After removing part of the BC from the column (water-leached), leaching continued with 0.05 M HCl at a 1:10 (BC:acid w/w) ratio with a flow rate of 20–30 ml hr^-1^ for 1 day to produce “acid-leached” BC. During the leaching, water and subsequently HCl were pumped through the vertical columns from the bottom upwards. Pumping stopped temporarily when leachate appeared on the top of the column and resumed after 2 days (in the case of water) or 1 day (in the case of HCl). A peristaltic pump was used to control the flow rate. Both water- and acid-leached BCs were oven-dried at 40°C for 3 days resulting to a moisture content of 13 and 6%w/w, respectively. Prior to mixing with the soil, the BCs (both untreated and leached) were ground and passed through a 0.5 mm sieve. Despite possible release of fresh materials after grinding of leached BCs to ≤ 0.5 mm, the pH measured in soil-leached BC slurries before incubation (Table 2) was lower than in slurries with untreated BC, hence satisfying the purpose of reducing or removing alkalizing effect of BC. Cations, anions and dissolved organic C removed by leaching with water and acid, respectively, can be found in S2 File.

**Table 2:** Mean soil slurry pH after treatment with various doses of the amendments at the start and end of incubation.

Soil	Amendment	Soil pH at the start of incubation						Soil pH at the end of incubation					
**Lampung soil**	**Cacao shell BC doses (%)**	**0**	**1**	**2**	**5**	**10**	**SE**	**0**	**1**	**2**	**5**	**10**	**SE**
	Untreated	4.0	6.3	6.8	7.6	8.4	0.1	5.7	6.9	7.6	8.3	9.0	0.2
	Water leached	4.0	-	5.7	6.6	7.2	0.0	5.8	-	6.3	7.1	7.9	0.3
	Acid leached	4.0	-	5.0	6.1	6.6	0.1	5.6	-	5.3	6.4	6.9	0.2
	**Rice husk BC doses (%)**	**0**	**1**	**2**	**5**	**10**	**SE**	**0**	**1**	**2**	**5**	**10**	**SE**
	Untreated	4.0	4.2	4.4	4.9	5.5	0.0	5.9	5.9	6.1	6.2	6.2	0.4
	Water leached	4.0	-	4.4	4.6	5.0	0.0	5.8	-	6.2	6.5	6.0	0.3
	Acid leached	4.0	-	3.9	3.6	3.3	0.0	6.2	-	5.4	5.7	4.7	0.4
	**Uncharred cacao shell doses (%)**	**0**	**1**	**2**	**5**	**10**	**SE**	**0**	**1**	**2**	**5**	**10**	**SE**
	Uncharred cacao shell	3.7	-	4.4	4.8	5.9	0.0	5.4	-	6.1	5.8	5.6	0.1
	**NaOH doses (ml)**	**0**	**0.35**	**1.25**	**1.8**	**-**	**SE**	**0**	**0.35**	**1.25**	**1.8**	**-**	**SE**
	NaOH	3.7	4.8	7.2	8.0	-	0.1	5.4	5.9	6.9	7.3	-	0.2
**Mkushi soil**	**Cacao shell BC doses (%)**	**0**	**1**	**2**	**5**	**10**	**SE**	**0**	**1**	**2**	**5**	**10**	**SE**
	Untreated	3.9	-	8.1	9.3	9.8	0.0	6.2	-	8.4	9.5	9.9	0.5
	Water leached	4.0	-	5.8	6.8	7.5	0.0	5.6	-	7.9	8.8	8.2	0.4
	Acid leached	4.0	-	-	6.5	6.8	0.1	5.6	-	-	8.5	8.4	0.5

SE is standard error calculated from all doses of each amendment for either start or end pH.

### Denitrification experiments

Air-dried soils were saturated with water and equilibrated to 5 kPa suction in a sand box (Eijkelkamp Agrisearch Equipment, Giesbeek, The Netherlands) over a 5 days period. Controlled pre-wetting was done to accommodate for the flush of microbial activity commonly observed upon rewetting of dry soil [44]. For the incubation assays, approx. 10 g sand box equilibrated soil was placed in 120 ml serum bottles together with a magnetic stirring bar. Treatments included BCs (untreated and leached) and uncharred cacao shell, the latter to assess the effect of the feedstock alone (in Lampung soil only), at doses of 0, 1, 2, 5 and 10% (dry weight basis). Weight losses during leaching were implicitly corrected for since the same weights of the treated chars were used. To investigate if the effect of BC on denitrification and its gaseous reaction products was merely a pH effect, another set of experiments was run with Lampung soil in which soil pH was manipulated by adding 0.35, 1.25 and 1.80 ml of 0.1M NaOH prior to anoxic incubation. Dose of NaOH was decided based on the alkalizing effect of BC, e.g. 1.8 ml 0.1M NaOH was equivalent to 10% untreated cacao shell BC in Lampung soil. All treatments were prepared in triplicate. In preparation of soil slurries, 30 ml of a 2 mM KNO_3_ solution were added to the bottles thereby providing ample NO_3_^-^ for denitrification. After the amendment, the effective pH values in the soil slurries were measured by a pH meter (Orion 2 Star, Thermo Fisher Scientific, Fort Collins, CO, USA) after 0.5 hour of oxic stirring. Thereafter, bottles were tightly closed with rubber septa and aluminum crimp seals and flushed with He (99.999%, AGA Industrial Gasses, Oslo, Norway) by alternately evacuating and He-filling the bottles 5 times using an automated manifold. This was done under constant stirring to achieve close to fully anoxic conditions. Measurements of pH in the slurries were repeated at the end of the incubation. An oxic incubation was carried out independently to check for BC-induced toxicity or stimulation of microbial activity (measured as O_2_ consumption) (Figure A in S3 File).

### Incubation and data collection

All incubations were carried out in a water bath at 20°C (which is within optimal range for microbial activities [45]) under constant stirring to maintain equilibrium of gases between the soil slurry and the bottle headspace. We used a robotized incubation system similar to that described by Molstad, Dörsch [38] to monitor the kinetics of O_2_ depletion, CO_2_ production and N-gas accumulation (NO, N_2_O, N_2_) during denitrification. The system consists of a water bath connected to a cryostat, placed under the robotic arm of an autosampler (Combi Pal, CTC, Switzerland). The water bath can accommodate up to 30 stirred bottles which are pierced repeatedly (here five-hourly) by the hypodermic needle of the autosampler which is connected to a peristaltic pump transporting the gas sample to a gas chromatograph equipped with various detectors and further to an NO-chemiluminescence analyzer. Details of the incubation system and gas analysis can be found in Description B in S1 File.

### Data handling

Rates of gas production and consumption were corrected for sampling loss and dilution as described by Molstad, Dörsch [38]. Maximum induced denitrification rate was calculated as the slope of the steepest part of the accumulation curve given by the sum of all N-gas products. The N_2_O/(N_2_O+N_2_) product ratio was calculated as the area under the curve of N_2_O divided by the area under the curve of NO+N_2_O+N_2_ [29]. As a cut off, the maximum accumulation of N_2_ was used, usually coinciding with the complete exhaustion of N-oxides in the bottles. In the instances where N-oxides were not exhausted, the accumulation curves were integrated over the entire experimental period. As a measure of NO net production in denitrification, maximum dissolved NO (nM) was calculated from headspace concentrations, using Henry’s law.

Statistical analysis was carried out using the R software [46]. Progression of denitrification was inspected by plotting cumulative N-gas and CO_2_ production as well as depletion of residual O_2_ over time. Maximum induced denitrification rates for each of the amendment type across its doses were subjected to one-way ANOVA and mean values of the doses were separated using Tukey’s Test at 5% significance level to establish statistically significant differences between BC doses.

To identify the possible factors explaining the effect of the amendments on maximum induced denitrification rate, N_2_O/(N_2_O+N_2_) ratio and maximum NO production, analyses of covariance (ANCOVA) were carried out. Firstly, ANCOVA was used to assess the effect of different types of untreated BC and doses, which was then followed by inclusion of BC leaching (untreated, water-leached and acid-leached) and effective pH in the statistical model as explanatory variables. Secondly, ANCOVA was used to separate the effect of labile C and other factors in BC on rate, N_2_O/(N_2_O+N_2_) ratio and maximum NO production by comparing charred and uncharred cacao shell. Furthermore, the effect of labile C and pH increase after adding BC on rate, N_2_O/(N_2_O+N_2_) ratio and maximum NO production were separated by comparing uncharred cacao shell and NaOH treatments using ANCOVA. pH, being an important explanatory variable for BC effect on N_2_O/(N_2_O+N_2_) ratio and maximum NO production, its values at the beginning and end of incubation are also presented.

## Results

### Effect of biochar on soil pH before and after anoxic incubation

**Fig 1:**
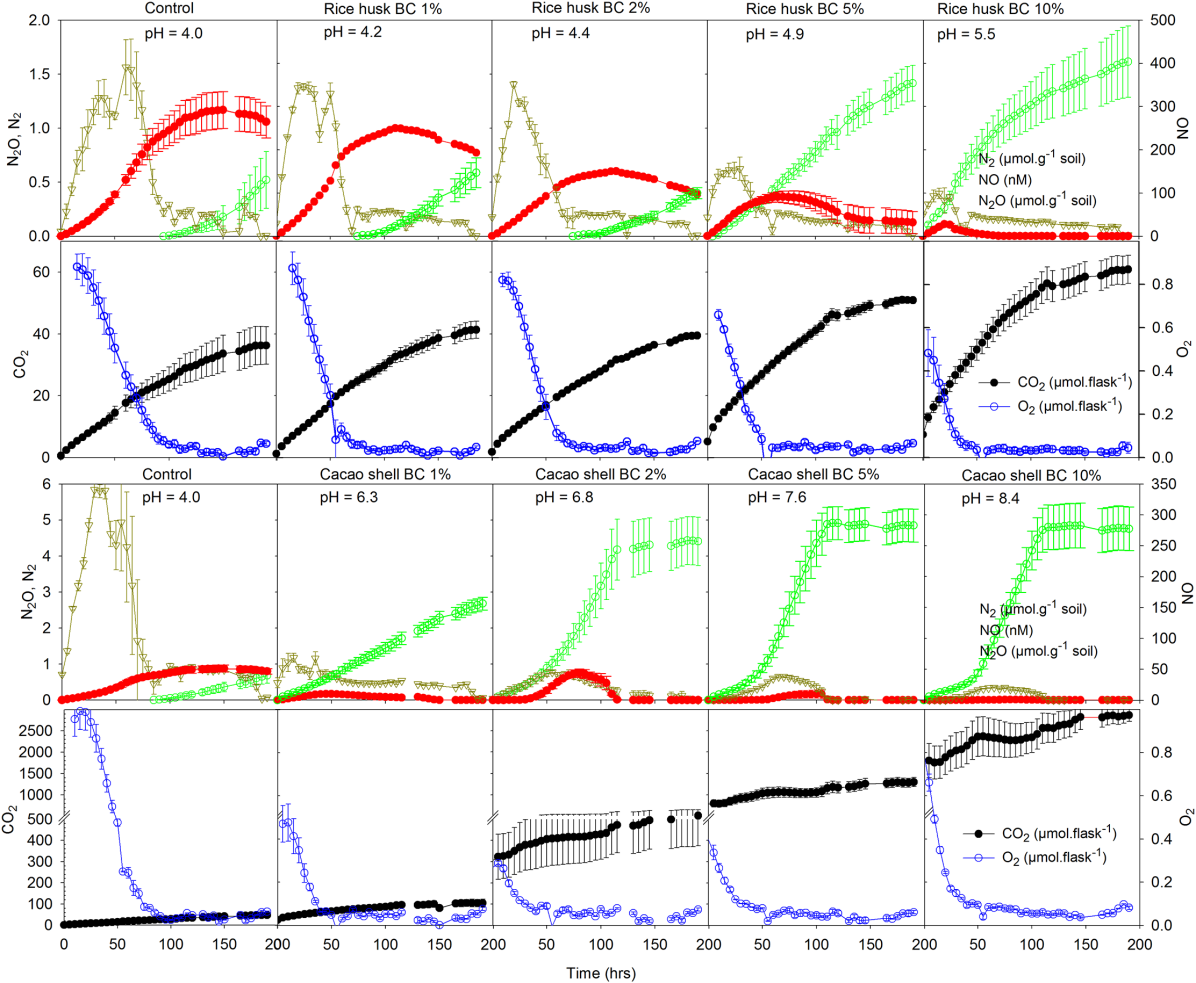
Denitrification kinetics and CO_2_ and O_2_ concentrations in anoxic incubations of Lampung soil amended with increasing doses of untreated rice husk BC (upper 2 panels) and cacao shell BC (lower 2 panels). Shown are averages of three incubations; error bars denote SE. Approximately 6.1 μmol NO_3_^-^-N g^-1^ was added to 9.8 g soil in the bottles. Note the differences in the scale of y-axis.

The addition of BC increased the pH of both soils (Table 2). The dose-dependent pH rise was more pronounced in Mkushi soil than in Lampung soil, reflecting the weaker buffer capacity (lower CEC) of the former (Table 1). Biochar from cacao shell and rice husk differed vastly in alkalinity and thus in its alkalizing effect on soil. For instance, addition of 1% (w/w) cacao shell BC to Lampung soil increased the soil pH by 2.3 units, whereas adding the same amount of rice husk BC resulted in only 0.2 units pH increase. Carbonate contributed a large part to the alkalizing effect of cacao shell BC as shown by high CO_2_ concentrations immediately following mixing the BC with acid soils (Fig 1).

Water leaching removed 159 cmol_c_ of base cations kg^-1^ (S2 File) from cacao shell BC and reduced its pH(H_2_O) from 9.8 to 9.6. Additional leaching with acid removed another 61 cmol_c_ of base cations and reduced its pH(H_2_O) to 8.0. For rice husk BC, water leaching removed 15cmol_c_ kg^-1^ base cations and reduced the pH from 8.4 to 8.2. Acid leaching removed an additional 19 cmol_c_ kg^-1^ and effectively acidified the BC (pH 2.5). In terms of mass, leaching with water and acid removed materials of approx. 65 and 14 mg g^-1^, respectively, of cacao shell BC and 7 and 5 mg g^-1^, respectively, of rice husk BC, and increased the surface area of BC (Table 1). For both BCs, base cations, in particular K^+^, removed by sequential water and acid leaching exceeded ammonium acetate exchangeable amounts (Table 1). The leaching treatment removed a significant part of the alkalizing effect of both BCs in soil (Table 2) and it may have changed other properties of BC. The cacao shell feedstock increased soil pH only modestly compared to its BC, if applied at an equivalent dose of mass (Table 2).

Anoxic incubation of soil slurries caused an increase in soil pH from initial values between 4.0 and 9.8 to final values between 5.4 and 9.9 (Table 2). In control soils and acidic soil-BC slurries, the pH increased more strongly than in alkaline slurries. This increase in pH can be attributed to denitrification (an alkalizing process), continuous release of cations from the BCs and exchange reactions during stirring.

### Kinetics of denitrification

Fig 1 and Figure B in S3 File show the kinetics of N-gas production and consumption together with the depletion of residual O_2_ (after He-flushing) and cumulative CO_2_ production (total inorganic carbon) in response to addition of untreated BC to Lampung and Mkushi soil, respectively. Controls (no BC addition) showed transient NO accumulation, instantaneous N_2_O net production and measurable N_2_ production after ~100 hours of incubation. Maximum NO accumulation was one order of magnitude greater in the Lampung soil (0.3–0.5 μM, Fig 1) than in Mkushi soil (0.05 μM, Figure B in S3 File).

Both BCs suppressed the net production of NO and N_2_O and increased N_2_ production, but cacao shell BC (Fig 1; lower panel) stimulated overall denitrification (measured as total N_2_ accumulation) more than rice husk BC (Fig 1; upper panel). With cacao shell BC doses > 2%, N_2_ production reached a plateau after slightly more than 100 hours incubation, indicating that all N-oxides were exhausted. In this case, cumulative N_2_ production roughly balanced the sum of initially present total soil N and added NO_3_^-^. Biochar also shortened the time needed to detect measurable N_2_ production (except in the 10% cacao shell BC addition to Mkushi soil), indicating earlier induction of N_2_O reductase (N_2_OR) activity in the presence of BC. In Lampung soil, the suppression of NO and N_2_O and stimulation of N_2_ as well as CO_2_ production was dose-dependent irrespective of BC type. In Mkushi soil, 2% cacao shell BC addition stimulated complete denitrification resulting in high production rates of N_2_ and practically eliminated N_2_O accumulation (Figure B in S3 File). However, with further increases in the dose of cacao shell BC, slurry pH increased up to pH 9 in this weakly buffered soil and maximum NO accumulation and N_2_ production decreased, indicating inhibition of denitrification at high pH. N_2_O suppression with concomitant increase in N_2_ production was also seen in the NaOH treatments of Lampung soil (Figure C in S3 File) and in incubation of BC in 2 mM KNO_3_ without soil (Figure D in S3 File). In contrast, uncharred cacao shell stimulated overall denitrification strongly, while suppression of N_2_O was small (Figure C in S3 File).

Water-leached rice husk BC caused only a modest decline in pH and resulted in denitrification kinetics similar to those with untreated BC in Lampung soil (compare Fig 1 and Figure E in S3 File). By contrast, addition of acid-leached rice husk BC reduced soil pH, but left the net production of N_2_O and overall N-gas largely unchanged when compared with the control soil (Figure F in S3 File). Unlike acid-leached rice husk BC, acid-leached cacao shell BC retained some of its N_2_O suppressing effect in Lampung soil (Figure F in S3 File) in line with its remaining alkalizing effect. However, the N_2_O suppressing effect of water or acid-leached cacao shell BC was non-linear with maximum suppression already reached at 2% BC. At higher doses of leached cacao shell BC, no further N_2_O suppression occurred and we observed biphasic kinetics in particular of NO accumulation showing two peaks during incubation (Figures E and F in S3 File).

Table 3 shows maximum induced denitrification rates for Lampung and Mkushi soil amended with rice husk and cacao shell BC, uncharred cacao shell and NaOH. In Lampung soil, addition of more than 2% untreated cacao shell BC significantly increased denitrification rates compared to the control (P˂0.05), whereas rice husk BC did not. Water- and acid-leaching of the cacao shell BC removed most of the stimulating effect. Higher doses of acid-leached rice husk BC caused a small but significant decrease in denitrification rate in Lampung soil (P˂0.05). In Mkushi soil, only 2% untreated cacao shell BC stimulated denitrification whereas leached BC did not. This contrasts findings from aerobic incubations, which showed clear stimulation of respiration by all doses of untreated BCs in both soils (Figure A in S3 File). NaOH also stimulated denitrification (Table 3) but to a much lesser extent compared to untreated cacao shell BC and uncharred cacao shell despite similar increases in soil pH (Table 2).

**Table 3:** Maximum inducible denitrification rates in Lampung and Mkushi soil amended with cacao shell BC, rice husk BC, uncharred cacao shell and NaOH.

Soil	Amendment	Denitrification rates^a^ (nmol N g^-1^ soil hr^-1^)					
**Lampung soil**	**Cacao shell BC doses (%)**	**0**	**1**	**2**	**5**	**10**	**SE**
	Untreated	22.7a	38.5ab	114.1bc	157.3c	116.3bc	27.7
	Water leached	26.3a	-	25.0a	38.5b	49.5c	2.1
	Acid leached	36.9a	-	20.8b	25.2b	37.2a	2.2
	**Rice husk BC doses (%)**	**0**	**1**	**2**	**5**	**10**	**SE**
	Untreated	28.5a	29.58a	16.8a	29.3a	31.0a	5.4
	Water leached	26.3a	-	25.6a	20.1a	17.0a	3.5
	Acid leached	33.1a	-	25.7a	16.5b	16.6b	2.7
	**Uncharred cacao shell doses (%)**	**0**	**1**	**2**	**5**	**10**	**SE**
	Uncharred cacao shell	36.3a	-	146.9b	209.7c	262.0d	4.7
	**NaOH doses (ml)**	**0**	**0.35**	**1.25**	**1.80**	**-**	**SE**
	NaOH	36.3ab	24.9a	48.6b	94.2c	-	5.3
**Mkushi soil**	**Cacao shell BC doses (%)**	**0**	**1**	**2**	**5**	**10**	**SE**
	Untreated	13.8a	-	35.9b	17.6a	12.0a	4.7
	Water leached	13.8a	-	17.8a	14.3a	8.6a	4.1
	Acid leached	13.8a	-	-	11.2a	12.6a	2.2

^a^Mean rate of various doses of each amendment in a row followed by different letters denote significant difference (Tukey’s test, P˂0.05). SE is standard error calculated from all doses of each amendment.

### Possible factors contributing to the BC effect on net N_2_O and NO production and denitrification rate

Linear model ANCOVA showed differences in the response of denitrification product ratio (N_2_O/(N_2_O+N_2_)), maximum NO accumulation and denitrification rate to BC type (rice husk or cacao shell) and dose, total C content (at onset of the experiment) and pH of the slurry (Table 4). In particular, BC type was a very important factor (p = 0.000). Doses were also important (p = 0.000 for denitrification product ratio and maximum NO accumulation; p = 0.01 for denitrification rate). Upon incorporation of BC leaching (untreated, water- and acid-leached BC) and pH as factors in addition to BC type and doses in the analysis, N_2_O/(N_2_O+N_2_) ratio, maximum NO accumulation and denitrification rates were significantly affected by all the factors at p = 0.000 (except the effect of BC dose on denitrification rate, which was at p = 0.003). Several interaction terms between factors were also significant (p<0.05).

**Table 4:** Results from stepwise linear ANCOVA showing the importance of labile C and pH for BC effect on denitrification rate, product ratio and maximum NO accumulation.

Analysis	Factors and interactions	N_2_O/(N_2_O+N_2_)	Rate	NO
BC effect (Cacao shell & rice husk BC)	BC type	***	***	***
	BC dose	***	*	***
	BC type:BC dose	*	*	ns
BC leaching (Cacao shell & rice husk BC either untreated, water-leached or acid-leached)	BC type	***	***	***
	BC leaching	***	***	***
	BC dose	***	**	***
	pH	***	***	***
	BC type:BC leaching	***	***	***
	BC type:pH	ns	***	.
	BC type:BC dose	***	ns	***
	BC leaching:BC dose	ns	ns	***
	BC leaching:pH	*	*	***
	BC dose:pH	**	ns	***
	BC type:BC leaching:BC dose	***	ns	***
	BC type:BC leaching:pH	ns	ns	ns
	BC type:BC dose:pH	*	ns	ns
	BC leaching:BC dose:pH	ns	ns	*
	BC type:BC leaching:BC dose:pH	*	ns	ns
Labile C effect (labile C vs other factors in cacao shell)	Cacao shell (BC & uncharred)	***	***	***
	C added	ns	**	***
	pH	***	***	***
	Cacao shell:pH	**	***	***
	Cacao shell:C added	*	.	***
	C added:pH	ns	ns	ns
	Cacao shell:pH:C added	ns	ns	**
pH effect (separate pH from labile C)	Material (NaOH & uncharred cacao)	.	***	**
	pH	***	***	***
	C added	ns	***	***
	Material:pH	ns	***	ns
	pH:C added	ns	***	ns

Signif. codes: 0 ‘***’ 0.001 ‘**’ 0.01 ‘*’ 0.05 ‘.’ 0.1 ‘ns ‘ 1, ‘:’ means interaction of factors.

ANCOVA also showed that total organic C (either as cacao shell or as its BC) added to the system was important in determining denitrification rate (p = 0.006) and maximum NO accumulation (p = 0.000) but not N_2_O/(N_2_O+N_2_) ratio (p = 0.41). In addition, a comparison of treatments with uncharred cacao shell, providing significant amounts of labile C, and NaOH, without addition of labile C, showed the strong influence of labile C on denitrification rate (p = 0.000) but not on N_2_O/(N_2_O+N_2_) ratio (p = 0.06). In this comparison, pH significantly affected both denitrification rate and N_2_O/(N_2_O+N_2_) (p = 0.000).

### NO accumulation and N_2_O/(N_2_O+N_2_) product ratios

**Fig 2:**
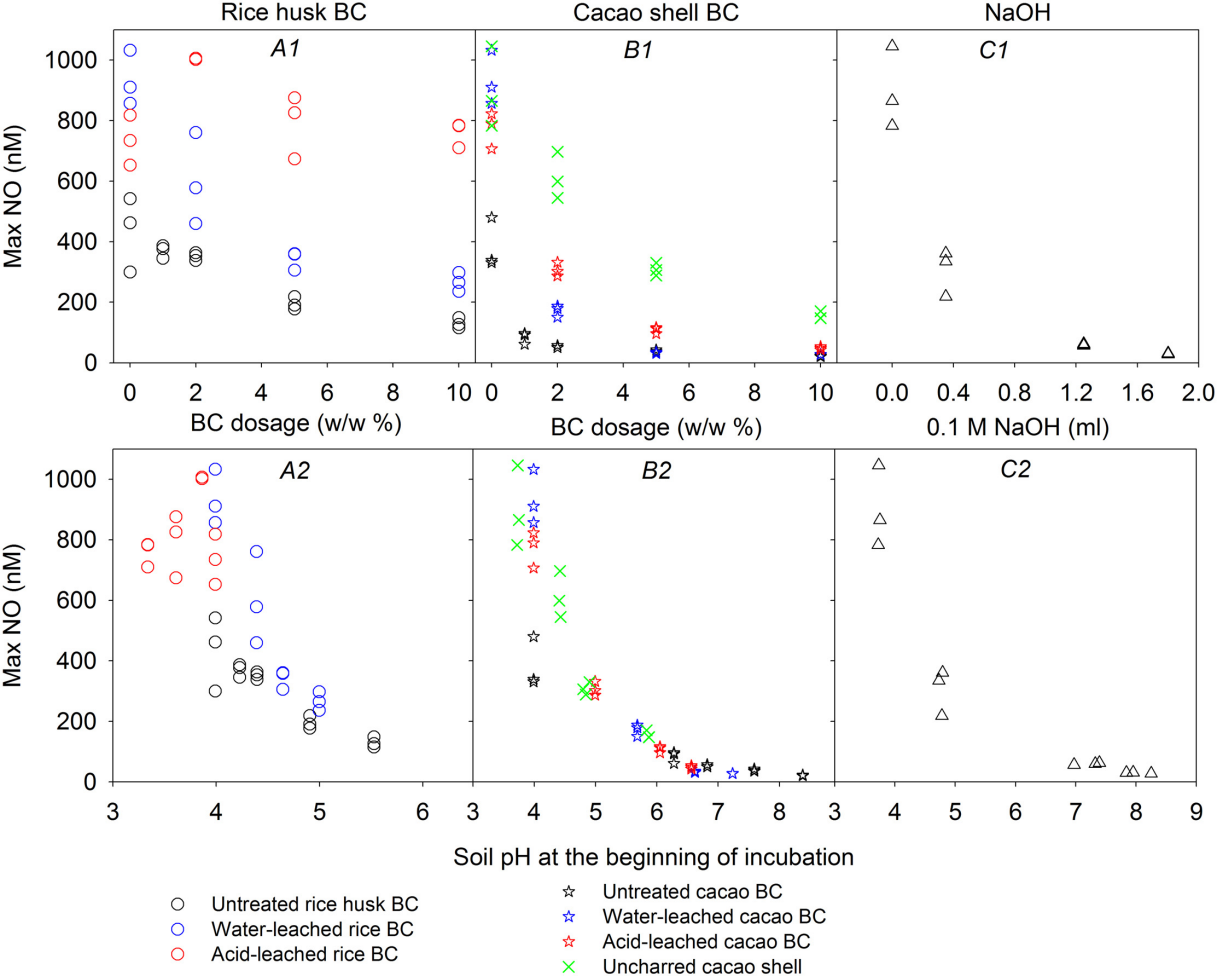
Maximum NO concentration in the liquid phase plotted against doses of BC, uncharred cacao shell and NaOH added to Lampung soil (upper panel—A1, B1 & C1), and against initial pH for Lampung soil amended with BC, uncharred cacao shell and NaOH (lower panel—A2, B2 & C2).

Increasing doses of both untreated rice husk and cacao shell BC, as well as NaOH, caused maximum NO accumulation to decrease (Fig 2 upper panel). Corresponding doses of leached BC reduced suppression of maximum NO accumulation. Acid leaching of rice husk BC entirely eliminated the suppression of NO accumulation. Uncharred cacao shell had weaker effect on suppression of NO accumulation than corresponding doses of cacao shell BC whether leached or not. Maximum NO accumulation decreased asymptotically with increasing pH to trace levels at pH > 6.5 (Fig 2 lower panel). The NO accumulation rate was greatest at the beginning of the incubation reaching maximum values within 72 hours (Fig 1 and Figures C, E and F in S3 File), except in Mkushi soil with > 5% cacao shell BC (Figure B in S3 File). Here NO accumulation gradually increased throughout the incubation period.

**Fig 3:**
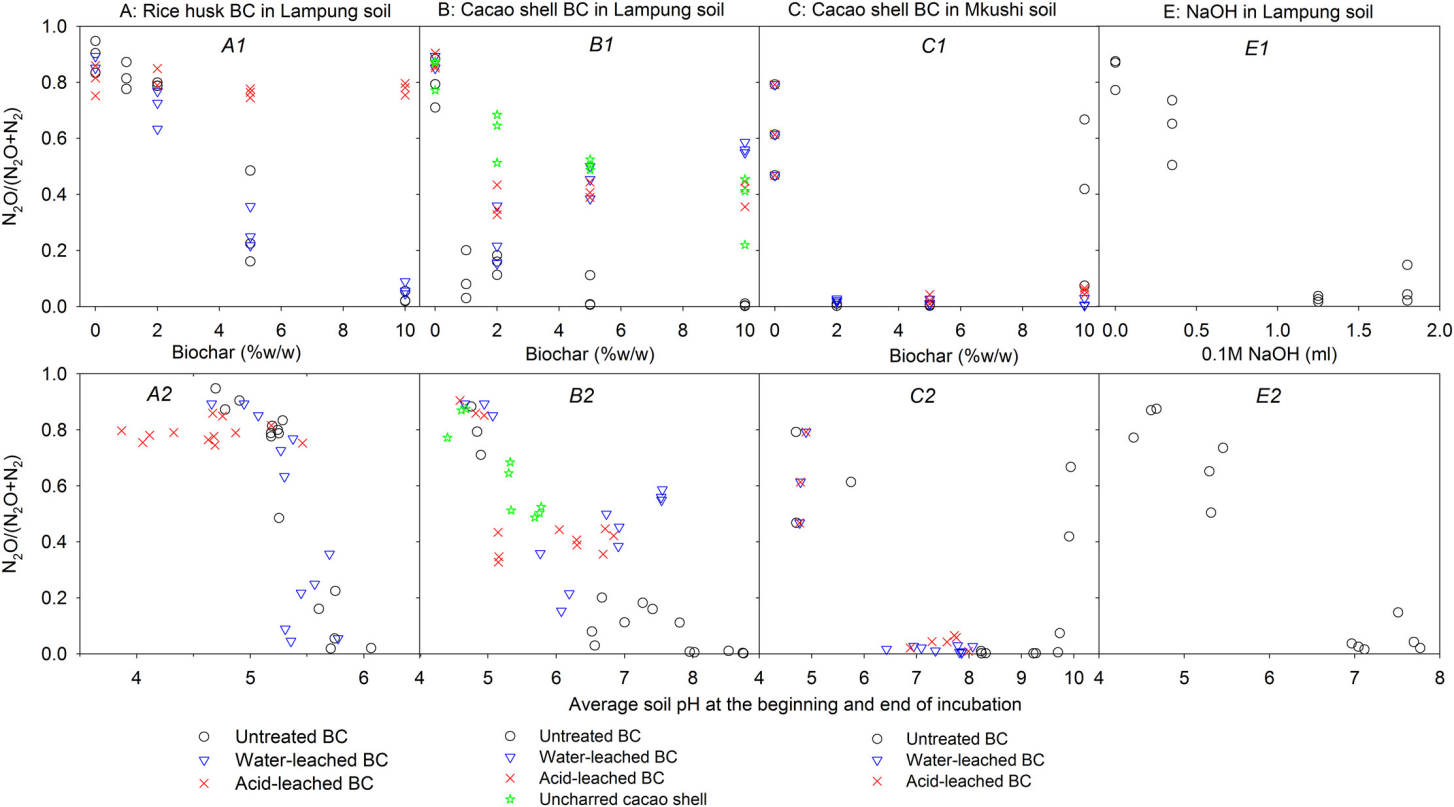
Plots of N_2_O product ratio of denitrification against BC dose (upper panel—A1, B1, C1 & D1) and against average effective soil pH (lower panel—A2, B2, C2 & D2) of BC, uncharred cacao shell and NaOH amended soil.

The N_2_O/(N_2_O+N_2_) product ratio decreased with increasing doses of untreated BC (Fig 3 upper panel). Rice husk BC addition to Lampung soil resulted in a decrease of the N_2_O/(N_2_O+N_2_) ratio with increase in dose, reaching values below 0.1 at 10% BC addition (Fig 3A1). Adding the same amounts of cacao shell BC to Lampung soil suppressed the denitrification product ratio much more strongly; reaching low product ratios already with 1% addition and increasing the doses did not have additional benefit in suppressing N_2_O. Cacao shell BC with its strong alkalizing effect was more effective in suppressing N_2_O than its feedstock at equivalent doses of mass (Fig 3B1). Thus, the strong effect of cacao shell BC compared to that of rice husk BC on the N_2_O product ratio could be linked to its strong alkalizing effect, resulting in greater soil pH increase at equivalent doses (Table 2, Fig 3). Due to its strong alkalizing effect, no N_2_O/(N_2_O+N_2_) data are available for cacao shell BC-amended Lampung soils in the pH range 4.8–6.6 (Fig 3B2). Therefore, our data do not allow a direct comparison of pH-related effects of the two BCs. In Mkushi soil, the N_2_O/(N_2_O+N_2_) ratio was reduced to zero even at the lowest dose (here 2%, which increased soil pH to 8.3; Fig 3C). A 10% cacao shell BC addition to Mkushi caused high, but uncertain values of product ratio probably due to suppression of overall denitrification activity (Figure B in S3 File). Thus, our data for BC-amended soils indicate that the N_2_O/(N_2_O+N_2_) ratio decreased from close to 1 at pH < 4 (no induction of N_2_OR activity) to close to zero at pH > 6 (sufficient induction of N_2_OR to prevent significant net production of N_2_O).

Addition of NaOH also decreased net N_2_O production (Fig 3D). In the pH range 4 to 7, the relationship between pH and N_2_O/(N_2_O+N_2_) product ratio had a significantly smaller slope for NaOH-amended- than for BC-amended soils but similar to that of uncharred cacao shell (Table 5).

**Table 5:** Regression coefficients of N_2_O product ratios explained by dose effect (w/w %) or by pH effect of different amendments added to Lampung soil.

Analysis	Amendment	Intercept	Slope	Significance of slope	R^2^
Dose effect	Untreated rice husk BC	0.90 (0.05)	-0.092 (0.010)	Slope different from zero (p<0.001)	0.91
	Water leached rice husk BC	0.84 (0.05)	-0.083 (0.010)	Slope not different from untreated rice husk BC (p>0.05)	
	Acid leached rice husk BC	0.81 (0.05)	-0.004 (0.010)	Slope different from untreated rice husk BC (p<0.001)	
	Uncharred cacao shell	0.77 (0.05)	-0.044 (0.010)	Slope different from untreated rice husk BC (p<0.001)	
pH effect	NaOH	2.34 (0.27)	-0.326 (0.047)	Slope different from zero (p<0.001)	0.80
	Untreated rice husk BC	5.12 (0.61)	-0.856 (0.113)	Slope different from NaOH (p<0.001)	
	Water leached rice husk BC	4.72 (0.81)	-0.797 (0.150)	Slope different from NaOH (p<0.01)	
	Acid leached rice husk BC	1.77 (0.52)	0.005 (0.107)	Slope different from NaOH (p<0.01)	
	Uncharred cacao shell	2.66 (0.59)	-0.399 (0.110)	Slope not different from NaOH (p>0.05)	

Intercept = value of product ratio at 0% BC and uncharred cacao shell addition or if pH of the soil would be zero. Slope = unit decrease in product ratio per percent increase of BC or uncharred cacao shell added or per unit increase in soil pH due to amendment added. Numbers in brackets are the standard errors.

Applying water-leached rice husk BC to Lampung soil resulted in a similar relationship between N_2_O/(N_2_O+N_2_) ratio and dose (or pH) as observed in soils with untreated rice husk BC (Fig 3A). Addition of acid-leached rice husk BC, which had lost all its alkalizing effect, resulted in large N_2_O/(N_2_O+N_2_) product ratios independent of BC dose (Fig 3A). Water and acid leached cacao shell BC decreased the N_2_O/(N_2_O+N_2_) ratio at low dose (2%), albeit less than untreated BC. At higher doses of leached BC, the ratios were relatively high compared to those of in response to untreated cacao shell BC additions at similar doses (Fig 3B).

N_2_O reduction to N_2_, which requires functional N_2_OR, only occurred after dissolved NO concentrations decreased to values ≤ 100 nM (Fig 1). In addition, N_2_O reduction only occurred at soil pH ≥ 5. At pH ≥ 5, e.g. after the amendment of rice husk BC at 5%, N_2_OR activity started immediately at the beginning of incubation (Fig 1). For soils or soil-BC mixtures with initial pH < 5 (e.g. treatments with 1–2% rice husk BC), denitrification driven alkalization, increasing pH to ~5 had to take place before induction of N_2_OR activity was observed. Initial delay in N_2_OR activity caused high accumulation of N_2_O in acidic soil or soil-BC mixtures.

## Discussion

### Effect of biochar on NO, N_2_O and N_2_ production and denitrification rate

Addition of untreated BCs to the two acidic Acrisols in this study suppressed the net production of both NO and N_2_O during anoxic incubation (Fig 1), which is in line with previously reported studies ([11] and references therein). Here we show that this suppression went along with increase in N_2_ production, suggesting increase in the activity of N_2_OR [47] due to alkalization [29].

Leaching of the BCs except for water-leached rice husk BC reduced or eliminated the effect of NO and N_2_O suppression (Figs 2 and 3), indicating that some of the BC constituents removed by leaching (S2 File) contributed to the suppression. Base cations and carbonates (shown by the high amount of CO_2_ released upon mixing of acidic soil with BC—Fig 1) were the major constituents removed by leaching, thus causing a decrease in alkalizing effect. Suppression of NO and N_2_O production in response to the addition of NaOH indicated that pH is an important factor contributing to the suppression. A recent study reported loss of alkalizing effect together with a loss in N_2_O suppression due to field aging of BC [48], suggesting that N_2_O suppression by BC might be a transient effect connected to the transiency of its alkalizing effect.

The N_2_O/(N_2_O+N_2_) product ratio decreased when the initial soil pH increased from pH 4 to 6 in response to the addition of BC (Fig 3). The rise in pH through addition of BC or NaOH removed the impairment of N_2_OR, typically seen at low pH [29, 33, 49]. The relief of this impairment through pH increase is similar to what has been reported for denitrifying pure cultures and for soils from long-term liming experiments in which raised pH stimulated N_2_OR and reduced N_2_O production or emissions [28, 29, 31, 33]. This direct effect of pH was attributed to a threshold pH above which functional N_2_OR is assembled [29, 31]. In this study, we found a threshold of pH ≈ 5 for the induction of N_2_OR based on the timing of N_2_ production onset (Fig 1), amount of accumulated denitrification intermediates (Fig 1) and pH at the beginning and end of incubation (Table 2). This threshold pH is close to threshold pH values for N_2_OR induction around pH 6, observed through detection of measurable N_2_ in earlier anoxic studies [29, 32]. The greater decrease of the N_2_O/(N_2_O+N_2_) ratio with increasing pH in rice husk BC-amended soil compared to that of previously published data [29, 32] and results from the NaOH-amended soil (Fig 3D and Table 5) suggest that BC has a somewhat stronger effect on the suppression of N_2_O than explained by pH alone. However, the effectiveness of N_2_O suppression seems to depend on the timing of induction of N_2_OR, which is controlled by the alkalizing effect of BC. Denitrification-driven alkalization contributed to induction of N_2_OR if the threshold pH for N_2_OR induction was not achieved by the BC alkalizing effect alone. Recently, Harter, Krause [50] reported an increased relative abundance of *nos*Z genes encoding for N_2_OR during 80 days of incubation after BC addition to soil, which is in line with the increased activity of N_2_OR observed in this study.

Only few recent studies have reported BC effects on NO production. Recently, Nelissen, Saha [37] reported a decrease in NO production similar to this study. The driver behind NO suppression by BC appears to be similar to that underlying N_2_O suppression because the two gases decreased with increasing doses of untreated BCs in a similar fashion (Fig 1). The concentration of the two gases increased initially and reached a peak before decreasing, although in all cases, NO reached the peak earlier than N_2_O. Low NO concentrations in BC- or NaOH-amended soils (Fig 1 and Figure C in S3 File) were likely due to the pH-increasing effect (Table 4), which prevents chemical decomposition of NO_2_^-^ to NO [51, 52], leaving only enzymatically produced NO to accumulate. Higher NO production in Lampung compared to Mkushi soil was probably due to higher microbial activities producing nitrite, part of which was decomposed chemically to NO at low pH. Our data also suggest that induction of N_2_OR is linked to low NO concentration, as N_2_OR activity was not initiated before NO concentration dropped to values below 100 nM. NO has been proposed to play an important role in the regulation of denitrification enzyme regulation [53], but little is known how reactive gaseous N species like NO react with BC.

In general, both aerobic and anaerobic respiration were stimulated by BC addition to soil (Fig 1 and Figure A in S3 File). Suppression of anaerobic respiration was only found at high doses of cacao shell BC added to Mkushi soil resulting in soil pH values > 9 (Figure B in S3 File). Anoxic incubation of untreated BC in 2 mM KNO_3_ solution without soil revealed that BC themselves carried out some denitrification activity which was expressed when residual O_2_ was fully exhausted (Figure D in S3 File). Interestingly, no N_2_O accumulated, suggesting full N_2_OR induction at high pH. Denitrification activity was clearly greater with rice husk (pH 8.4) than cacao shell BC (pH 9.8). This might reflect the inability of the denitrifier community to thrive when too much BC is added driving soil pH to high values at which NO_2_^-^ may accumulate to toxic levels [54]. Additionally, the osmotic effect of salts due to high dose (10%) BC in poorly buffered Mkushi soil may have inhibited microbial activity. Other than at high dose, our BC did not have any direct inhibitory effect on microbial activities such as shown for BC-mediated ethylene production [23].

BC is a complex material, which may alter many soil variables besides pH. In particular, BC increased bioavailable carbon (C) (Figure D in S3 File; residual O_2_ was consumed and CO_2_ was produced during incubation of BC without soil) [55, 56] and nutrients (S2 File) which could stimulate microbial growth [56] and affect the regulation of denitrification. Addition of bioavailable C clearly affected denitrification rate as seen after adding uncharred cacao shell (Tables 3 and 4), but it did not affect the product ratio (Table 4). The decrease in product ratio with increase in BC dose applied was better explained by pH increase than by C-addition in our ANCOVA. The contribution of bioavailable organic C and/or nutrients of cacao shell BC to increased denitrification rates is clearly seen when comparing cacao shell BC treatments with NaOH treatments at similar soil pH.

Leaching of BC, which mimics field aging, affected both its alkalinity and surface chemistry (Table 1 and S2 File). Changes to BC surface chemistry may occur through alterations of surface functional organic groups. The leaching experiments showed that certain BC types such as cacao shell BC may be more resistant to aging presumably through release of base cations and secondary carbonation, which would explain the relatively minor effect of acid leaching on cacao shell BC’s alkalinizing effect (Table 1 and S2 File). Denitrification experiments with leached cacao shell BC did not show ordinary dose response. Instead, higher doses of leached cacao shell BC resulted in conspicuous biphasic NO kinetics with two peaks in Lampung soil, a delayed peak of N_2_O production as well as delayed production of N_2_ by either enzymatic or chemical pathways (Figures E and F in S3 File) [57]. This went along with higher N_2_O/(N_2_O+N_2_) ratios at high doses as compared with untreated BC (Fig 3B). This may point at some chemical interaction of newly exposed BC surfaces with denitrification intermediates. Initially, leached cacao shell BC may have acted as electron sink [11, 18], competing with denitrification reductases for electrons. However, there was no indication of chemical reaction such as sorption and desorption between BC and N-compounds in an anoxic incubation of BC (untreated or leached) without soil (Figure D in S3 File).

### Factors determining NO and N_2_O suppression by biochar

In this study, we found that the pH effect of BC in acid soil played a major role in the suppression of both NO and N_2_O under anoxic conditions. However, any extrapolation of our data beyond acidic soils needs to be done with caution. Cayuela, Sánchez-Monedero [17] also observed reduced N_2_O/(N_2_O+N_2_) ratios during N_2_O peak emission in wet soils amended with brush BC but a direct pH effect was not clearly captured probably because of the small pH increase (0.1 pH units). Instead, Cayuela, Sánchez-Monedero [17] could show that the observed reduction in N_2_O/(N_2_O+N_2_) ratios were positively correlated to the buffer capacities of the added BC. Earlier, Yanai, Toyota [20] had concluded that suppression of N_2_O emissions (which they believed originated from denitrification) by BC was not the result of changes in soil chemical properties. Cayuela, Sánchez-Monedero [17] and the present study clearly show that BC can affect the soil chemical properties with consequences for the product stoichiometry of denitrification. In this study, we used controlled anoxia with direct quantification of N_2_ production to study the effect of BC on denitrification stoichiometry. Yanai, Toyota [20] did not separate N cycling processes and their study could have been confounded by nitrification, an acidifying process, as suggested by the decrease in pH at the end of their incubations. We did not account for dissimilatory nitrate reduction to ammonium (DNRA) in this study; however, it is unlikely that this process played a major role as we recovered the added nitrate quantitatively as N_2_.

The steeper slopes of N_2_O/(N_2_O+N_2_) versus pH in BC treatments compared to NaOH and uncharred cacao shell treatments (Table 5) indicate that some other factors may have contributed to the suppression of N_2_O in addition to the pH effect. The similarity of the slopes for uncharred cacao shell and NaOH suggests that stronger suppression of N_2_O by BC was not due to cacao shell itself or to labile C but to some other BC property. Biochar redox behavior (electron shuttling), where the electron-conductance of BC serves as a catalyst in denitrification as suggested by Cayuela, Sánchez-Monedero [17] could be one of these factors. The reduction or elimination of BC suppression of N_2_O after leaching of BC in this study raises questions about how leaching affects electron shuttling and how important electron shuttling is, in suppressing N_2_O.

## Conclusions

This study is the first of its kind assessing BC effects under full denitrification conditions, simultaneously quantifying NO, N_2_O and N_2_ production at high temporal resolution. We found compelling evidence that BC strongly suppresses relative NO and N_2_O net production from denitrification in two acid soils, resulting in a reduced propensity for NO and N_2_O emissions. Increase of soil pH by BC addition was identified as a major factor mediating this suppression. NO suppression was linked to less chemical decomposition of NO_2_^-^ to NO due to pH increase. N_2_O suppression on the other hand was in accordance with the notion that raising pH in acid soils greatly stimulates N_2_OR activity resulting in more complete denitrification with N_2_ as the dominating end product. Other factor(s) contributing causally to the observed increase in N_2_OR activity cannot be excluded and need further testing.

## Supporting Information

S1 File Description of biochar production, incubation system operation and gas chromatograph detectors. Biochar production (Description A). Incubation system operation and gas chromatograph detectors (Description B).
(DOCX)

S2 File Constituents removed from BC through leaching. Constituents removed from BC through leaching with water and strong acid (HCl) (Table A).
(DOCX)

S3 File Mean oxygen consumption during oxic incubations and kinetics of gas production (N_2_, N_2_O, NO, CO_2_) and consumption (O_2_) during anoxic incubations. Mean oxygen consumption in BC amended soils during oxic incubations (Figure A). Denitrification kinetics and CO_2_ and O_2_ concentrations in incubations of Mkushi soil amended with untreated cacao shell BC (Figure B). Denitrification kinetics and CO_2_ and O_2_ concentrations in incubations of Lampung soil amended with uncharred cacao shell (upper 2 panels) and 0.1M NaOH (lower 2 panels) (Figure C). Denitrification kinetics and CO_2_ and O_2_ concentrations in anoxic incubations of 2.36 g BC without soil in 30 ml 2mM KNO_3_ (Figure D). Denitrification kinetics and CO_2_ and O_2_ concentrations in incubations of Lampung soil amended with water-leached rice husk BC (upper 2 panels) and cacao shell BC (lower 2 panels) (Figure E). Denitrification kinetics and CO_2_ and O_2_ concentrations in incubations of Lampung soil amended with acid-leached rice husk BC (upper 2 panels) and cacao shell BC (lower 2 panels) (Figure F).
(DOCX)
