# Supplementary material for: Effect of Soil pH Increase by Biochar on NO, N2O and N2 Production during Denitrification in Acid Soils
Source: PLoS One. 2015 Sep 23;10(9):e0138781. doi: 10.1371/journal.pone.0138781 (PMC4580641; doi:10.1371/journal.pone.0138781)
Supplement: S1 File — Biochar production (Description A). Incubation system operation and gas chromatograph detectors (Description B). (DOCX) [file pone.0138781.s001.docx]

**S1 File. Description of biochar production, incubation system operation and gas chromatograph detectors**

**Description A. Biochar production**

The biochars (BCs) were produced in a locally fabricated metal kiln with a volume of 30-40L at a temperature of 250-350ºC for 3.5hours. The BC yield was 22% and 30.4% per unit weight of biomass. The schematic drawing of the kiln and a photograph from Hale et al., Chemosphere 2013, 57(11), 1612-1619 is shown below.


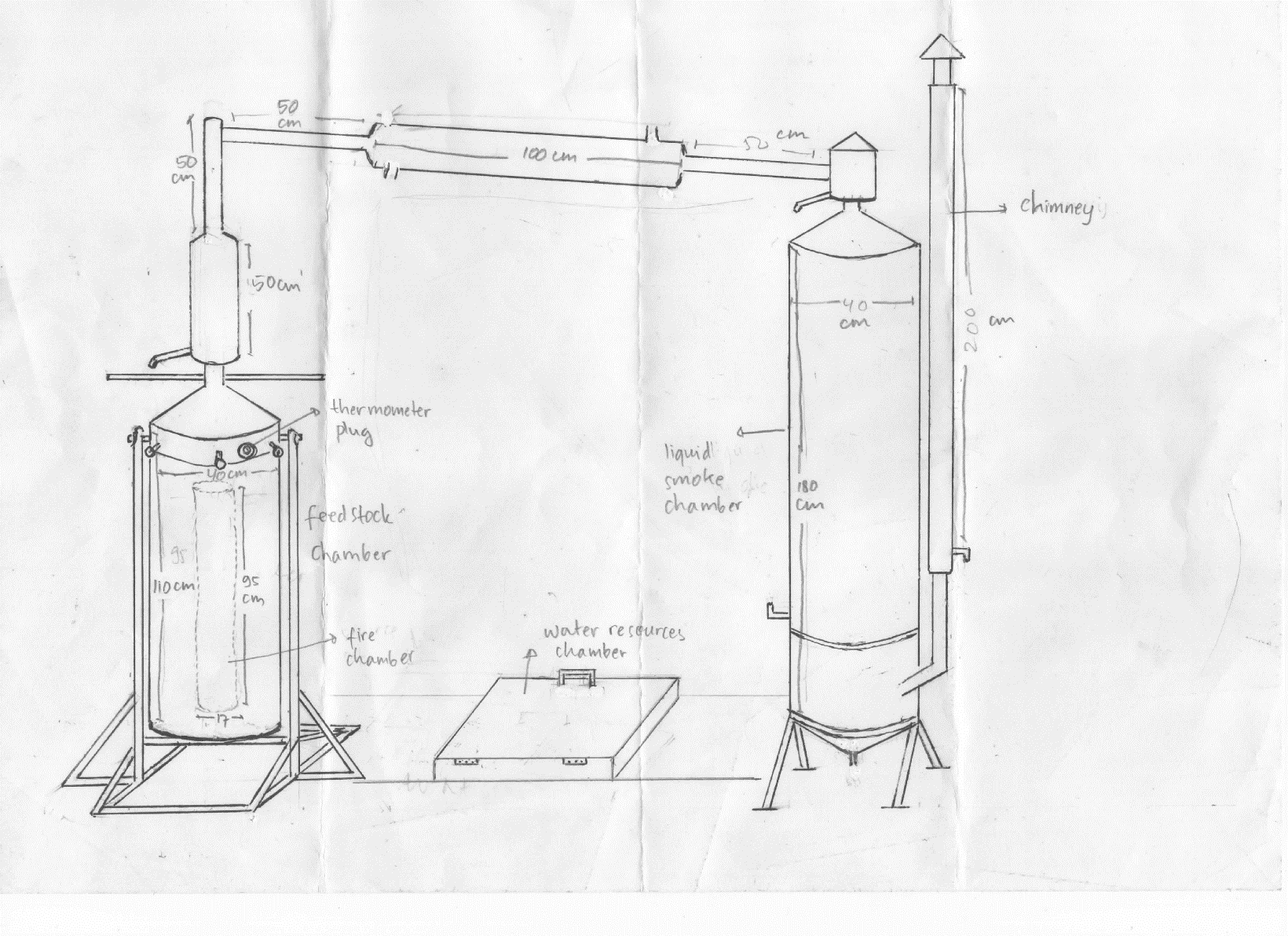

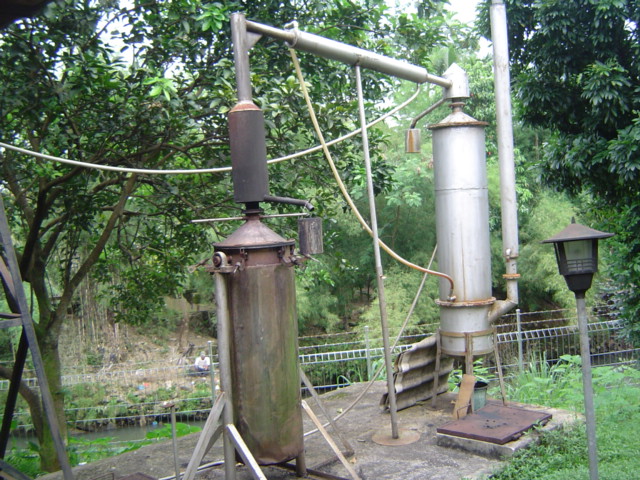


**Estimation of charring temperature by use of thermogravimetric analysis (TGA)**

**Stepwise TGA Analysis**: The degree of charring has been studied by measuring the weight loss of a char sample as it undergoes additional pyrolysis under a nitrogen atmosphere in a thermogravimetric analyzer (TGA). Each sample was heated sequentially to 8 increasing temperatures, varying by 100-degree increments, between 200 and 900^o^C, with a hold time of one hour at each temperature. The weight loss occurring at 200^o^C was taken as the moisture content of the sample. The weight loss at each temperature increment above 200^o^C, after correction for moisture and ash content, was used to determine the cumulative weight loss (total weight loss), as well as incremental loss profiles at each temperature step.

**Estimation of degree of charring:** The analysis of the data attempts to rank the chars by degree of charring using three measures taken from the stepwise analysis. The first measure is the total weight loss (sum of weight loss at each step from 300 to 900^o^C) after correction for moisture and ash content. The second measure is the ratio of high temperature weight loss (sum of weight loss from 600 to 900^o^C) to low temperature weight loss (sum of weight loss from 300 to 500^o^C). The third measure is the temperature of maximum weight loss. This should indicate that much of the sample has effectively seen a maximum charring temperature lower than this temperature.

The "degree of charring" is not meant to be an estimate of the highest heat treatment temperature seen by the char, since the degree of charring is a factor of both highest heat treatment temperature and the duration of charring. The comparison with the reference chars is meant to provide a scale to compare the test chars to and give an estimate of the "effective charring temperature".

**Reference chars:** The reference series of chars was produced by pyrolysis in a muffle furnace under inert (nitrogen) atmosphere for eight hours at the designated charring temperature. Temperatures were carefully controlled and varied by 50 or 100°C increments from 250°C to 900°C. The reference materials consisted of pine wood (*Pinus ponderosa*), switch grass (*Panicum virgatum*), and purified cellulose.

**Cacao and rice hull chars**: The cacao shell and rice hull char samples from Indonesia were provided by Gerard Cornelissen, Norges Geotekniske Institutt.

**Total weight loss**

The total weight loss of reference chars (in percentage) after correction for moisture and ash content are shown below. The numbers in parenthesis are standard deviations. (n=3 for pine wood char and n=2 for switchgrass chars)

| Charring  Temperature (ºC) | Pine wood  char | Switch grass  char | Cellulose  char |
| --- | --- | --- | --- |
| 250 | 64.3 (0.7) | 61.2 (2.90) | 48.9 |
| 300 | 48.7 (2.2) | 45.5 (2.03) | 44.8 |
| 350 | 37.0 (1.8) | 38.0 (2.04) | 43.1 |
| 400 | 34.0 (2.3) | 32.9 2.04) | 37.4 |
| 450 | 28.4 (2.6) | 25.5 (0.37) | 21.6 |
| 500 | 18.0 (0.6) | 22.0 (0.06) | 15.8 |
| 600 | 17.0 (2.0) | 18.2 (0.80) | 10.7 |
| 700 | 14.0 (1.9) | 14.7 (0.96) |  |
| 800 | 14.5 (3.0) | 14.5 (2.23) |  |
| 900 | 12.1 (1.8) | 13.1 (2.93) |  |

The total weight loss of the cacao shell char was 32.9% (Standard Deviation 3.6, n=3), which would indicate a charring temperature of about 400^o^C.

The total weight loss of the rice hull char was 33.1% (Standard Deviation 0.66, n=3), which would be the equivalent of an 8 hour charring temperature of about 400^o^C.

**High to Low temperature weigh loss ratio**

The ratio of high temperature weight loss (sum of weight loss from 600 to 900^o^C) to low temperature weight loss (sum of weight loss from 300 to 500^o^C) for the reference chars are shown below. The numbers in parenthesis are standard deviations. (n=3 for pine wood char and n=2 for switchgrass chars)

| Charring  Temperature (ºC) | Pine wood  char | Switch grass  char | Cellulose  char |
| --- | --- | --- | --- |
| 250 | 0.25 (0.02) | 0.23 (0.03) | 0.03 |
| 300 | 0.64 (0.11) | 0.46 (0.02) | 0.11 |
| 350 | 0.78 (0.11) | 0.64 (0.00) | 0.32 |
| 400 | 1.06 (0.23) | 0.96 (0.06) | 0.65 |
| 450 | 1.57 (0.24) | 1.42 (0.17) | 0.97 |
| 500 | 2.51 (0.10) | 2.20 (0.04) | 2.87 |
| 600 | 2.85 (0.18) | 2.65 (0.07) | 6.70 |
| 700 | 2.84 (0.05) | 2.48 (0.02) |  |
| 800 | 3.05 (0.15) | 2.23 (0.01) |  |
| 900 | 2.90 (0.08) | 2.04 (0.15) |  |

The ratio of high temperature weight loss to low temperature weight loss of the cacao shell char was 1.8 (Standard Deviation 0.08, n=3), which would indicate a charring temperature of between 450 and 500^o^C.

The ratio of high temperature weight loss to low temperature weight loss of the rice hull char was 1.4 (Standard Deviation 0.04, n=3), which would be the equivalent of an 8 hour charring temperature of between 450 and 500^o^C.

**Incremental loss profiles**

The incremental loss profile of the cacao shell char is shown below. The temperature of maximum weight loss of the cacao shell occurs at 700^o^C, indicating that the char has seen a maximum charring temperature lower than this temperature. The temperature of maximum weight loss of the rice hull char occurs at 600^o^C, indicating that the char has seen a maximum charring temperature lower than this temperature.


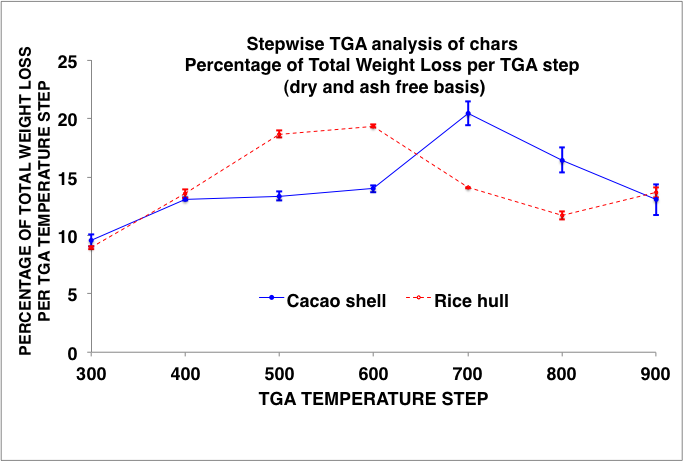


The TGA step with the maximum weight loss (degrees Celsius) of reference chars are shown below:

| Charring  Temperature (ºC) | Pine wood  char | Switch grass  char | Cellulose  char |
| --- | --- | --- | --- |
| 250 | 300 | 300 | 400 |
| 300 | 500 | 400 | 500 |
| 350 | 500 | 500 | 600 |
| 400 | 500 | 500 | 600 |
| 450 | 600 | 600 | 600 |
| 500 | 600 | 600 | 600 |
| 600 | 700 | 700 | 700 |
| 700 | 700 | 900 |  |
| 800 | 900 | 900 |  |
| 900 | 900 | 900 |  |

**Description B. Incubation system operation and gas chromatograph detectors**

The incubation system consists of a water bath connected to a cryostat, placed under the robotic arm of an autosampler (Combi Pal, CTC, Switzerland). Submersible magnetic stirrers (Variomag HP 15; H+P Labortechnik GmbH, Germany) accommodate up to 30 continuously stirred bottles and additional bottles with calibration gasses, which are pierced at selected intervals by the hypodermic needle of the programmable autosampler. For each sampling, an aliquot of the headspace gas (ca. 1.2 ml) is removed by a peristaltic pump (Gilson Minipuls 3) and transferred to a gas chromatograph (Model 7890A, Agilent, Santa Clara, CA, US) and an NO chemiluminescence analyzer (model 200A; Advanced Pollution Instrumentation) via dedicated sampling loops. He 5.0 was used as carrier gas. CO_2_ and N_2_O were separated by a 20 m wide-bore (0.53 mm diameter) Poraplot Q capillary column run at 38ºC and the bulk gases (N_2_, O_2_ and Ar) on a 30 m 5Å molsieve capillary column run at 25ºC. A packed Heysept column was used for back-flushing water. The analytical columns were connected to a thermal conductivity detector (TCD) and an electron capture detector (ECD) for quantification of N_2_O. The ECD was run at 375ºC with 17 ml min^-1^ Ar/methane (CH_4_) (90/10 vol%) as a make-up gas. The chemiluminescence NOx analyzer was programmed to analyze NO only and coupled to a dedicated sampling loop via an open split. After each sampling, the pump was automatically reversed, pumping back an equal amount of He into the incubation bottles to maintain atmospheric pressure. The resulting dilution (approx. 1.0% per injection) and the leakage of O_2_ and N_2_ into the bottles were estimated based on empty flasks with air and He, respectively. Bottles filled with certified standards were included into the sampling sequence for calibrating the incubation system.
