# Supplementary material for: Effect of Soil pH Increase by Biochar on NO, N2O and N2 Production during Denitrification in Acid Soils
Source: PLoS One. 2015 Sep 23;10(9):e0138781. doi: 10.1371/journal.pone.0138781 (PMC4580641; doi:10.1371/journal.pone.0138781)
Supplement: S2 File — Constituents removed from BC through leaching with water and strong acid (HCl) (Table A). (DOCX) [file pone.0138781.s002.docx]

**S2 File. Constituents removed from BC through leaching**

**Table A. Constituents removed from BC through leaching with water and strong acid (HCl)**

| Leaching procedure | Biochar constituents (cmol_c_ . kg^-1^ dry BC) | | | | | | | |  | | % (w/w) | |
| --- | --- | --- | --- | --- | --- | --- | --- | --- | --- | --- | --- | --- |
|  | Ca^2+^ | K^+^ | Mg^2+^ | Na^+^ | SO_4_^2-^ | NO_3_^-^ | Cl^-^ | PO_4_^3-^ | | DOC | | TN |
| Water leaching of cacao shell BC | 0.52 | 153.64 | 4.57 | 0.69 | 5.76 | 0.01 | 1.75 | 2.50 | | 0.02 | | 0.00 |
| Acid leaching of cacao shell BC | 17.54 | 22.39 | 20.89 | 0.21 | 0.30 | 0.00 | 3.19* | 0.24 | | 0.00 | | 0.00 |
| Total | **18.06** | **176.02** | **25.46** | **0.90** | **6.06** | **0.01** | **1.44*** | **2.75** | | **0.02** | | **0.01** |
| Water leaching  of rice husk BC | 0.17 | 14.04 | 0.62 | 0.35 | 0.85 | 0.00 | 0.92 | 2.19 | | 0.02 | | 0.00 |
| Acid leaching  of rice husk BC | 7.93 | 6.92 | 5.10 | 0.11 | 0.11 | 0.00 | 14.85* | 0.73 | | 0.00 | | 0.00 |
| Total | **8.09** | **20.95** | **5.72** | **0.45** | **0.95** | **0.00** | **13.92*** | **2.92** | | **0.02** | | **0.00** |

For details on water and acid leaching of BCs, see Materials and Methods. Total constituent is what was removed from acid leached BC. DOC – Dissolved organic carbon, TN – Total nitrogen. Cations were measured using ICP-OES (Perkin Elmer, USA), anions using Lachat IC5000 Ion Chromatograph w/XYZ Autosampler (Zellweger analytics, Inc., IL USA), DOC was analyzed using a Total Organic Carbon Analyzer (Shimadzu Corp., Japan), TN was analyzed using a CHN analyzer (CHN-1000, LECO USA). * means that Cl^-^ was added to BC from HCl during leaching experiment.
