# Supplementary material for: Effect of Soil pH Increase by Biochar on NO, N2O and N2 Production during Denitrification in Acid Soils
Source: PLoS One. 2015 Sep 23;10(9):e0138781. doi: 10.1371/journal.pone.0138781 (PMC4580641; doi:10.1371/journal.pone.0138781)
Supplement: S3 File — Mean oxygen consumption in BC amended soils during oxic incubations (Figure A). Denitrification kinetics and CO2 and O2 concentrations in incubations of Mkushi soil amended with untreated cacao shell BC (Figure B). Denitrification kinetics and CO2 and O2 concentrations in incubations of Lampung soil amended with uncharred cacao shell (upper 2 panels) and 0.1M NaOH (lower 2 panels) (Figure C). Denitrification kinetics and CO2 and O2 concentrations in anoxic incubations of 2.36 g BC without soil in 30 ml 2mM KNO3 (Figure D). Denitrification kinetics and CO2 and O2 concentrations in incubations of Lampung soil amended with water-leached rice husk BC (upper 2 panels) and cacao shell BC (lower 2 panels) (Figure E). Denitrification kinetics and CO2 and O2 concentrations in incubations of Lampung soil amended with acid-leached rice husk BC (upper 2 panels) and cacao shell BC (lower 2 panels) (Figure F). (DOCX) [file pone.0138781.s003.docx]

**S3 File. Mean oxygen consumption during oxic incubations and kinetics of gas production (N_2_, N_2_O, NO, CO_2_) and consumption (O_2_) during anoxic incubations**

Figure A. Mean oxygen consumption in BC amended soils during oxic incubations. Treatment mean is the average of 3 replicates with standard error. Treatment means followed by different letters are significantly different (P<0.05). Note different scaling of y-axis.

Figure B. Denitrification kinetics and CO_2_ and O_2_ concentrations in incubations of Mkushi soil amended with untreated cacao shell BC. Shown are averages of three incubations; error bars denote SE. Approximately 7.2 µmol NO_3_^-^-N g^-1^ was added to 8.3 g soil in the bottles.

Figure C. Denitrification kinetics and CO_2_ and O_2_ concentrations in incubations of Lampung soil amended with uncharred cacao shell (upper 2 panels) and 0.1M NaOH (lower 2 panels). Shown are averages of three incubations; error bars denote SE. Approximately 7.1 µmol NO_3_^-^-N g^-1^ was added to 8.4 g soil in the bottles.

Figure D. Denitrification kinetics and CO_2_ and O_2_ concentrations in anoxic incubations of 2.36 g BC without soil in 30 ml 2mM KNO_3_. Acid-leached BCs were spiked with N_2_O gas (0.1 ml at 1 atm pressure) at 65 hrs of incubation. Shown are averages of three incubations; error bars denote SE. Approximately 25.38 µmol NO_3_^-^-N g^-1^ BC was added to the bottles.

Figure E. Denitrification kinetics and CO_2_ and O_2_ concentrations in incubations of Lampung soil amended with water-leached rice husk BC (upper 2 panels) and cacao shell BC (lower 2 panels). Shown are averages of three incubations; error bars denote SE. Approximately 7.4 µmol NO_3_^-^-N g^-1^ was added to 8.1 g soil in the bottles.

Figure F. Denitrification kinetics and CO_2_ and O_2_ concentrations in incubations of Lampung soil amended with acid-leached rice husk BC (upper 2 panels) and cacao shell BC (lower 2 panels). Shown are averages of three incubations; error bars denote SE. Approximately 7.6 µmol NO_3_^-^-N g^-1^ was added to 7.9 g soil in the bottles.
